# Polymeric composite devices for localized treatment of early-stage breast cancer

**DOI:** 10.1371/journal.pone.0172542

**Published:** 2017-02-28

**Authors:** Kwabena Kan-Dapaah, Nima Rahbar, Wole Soboyejo

**Affiliations:** 1 Department of Biomedical Engineering, University of Ghana, Accra, Greater Accra, Ghana; 2 Department of Civil and Environmental Engineering, Worcester Polytechnic Institute (WPI), Worcester, MA, United States of America; 3 Department of Mechanical and Aerospace Engineering, Princeton University, Princeton, NJ, United States of America; 4 Princeton Institute for Science and Technology of Materials (PRISM), Princeton University, Princeton, NJ, United States of America; 5 Department of Mechanical Engineering, Worcester Polytechnic Institute (WPI), Worcester Polytechnic Institute, Worcester, MA, United States of America; 6 Department of Biomedical Engineering, Worcester Polytechnic Institute (WPI), Worcester, MA, United States of America; Brandeis University, UNITED STATES

## Abstract

For early-stage breast cancers mastectomy is an aggressive form of treatment. Therefore, there is a need for new treatment strategies that can enhance the use of lumpectomy by eliminating residual cancer cells with limited side effects to reduce local recurrence. Although, various radiotherapy-based methods have been developed, residual cells are found in 20–55% of the time at the first operation. Furthermore, some current treatment methods result in poor cosmesis. For the last decade, the authors have been exploring the use of polymeric composite materials in single and multi-modal implantable biomedical devices for post-operative treatment of breast cancer. In this paper, the concept and working principles of the devices, as well as selected results from experimental and numerical investigations, are presented. The results show the potential of the biomedical implants for cancer treatment.

## 1 Introduction

Breast cancer is the most frequently occuring cancer among females in the world. [1] It accounted for 512, 900 deaths worldwide out of 1.7 million new cases in 2012 (15% of all cancer deaths among females and 25% of all cancer cases). [1] Advances in detection techniques have led to the implementation of screening programmes, resulting in increased detection of early breast carcinomas. [2] For such small breast cancers, mastectomy is an aggressive form of treatment. Lumpectomy, followed by adjuvant whole breast radiotherapy (WBRT) to kill residual breast cancer cells has been a standard treatment strategy. However, issues related to treatment schedules of WBRT imposes a lot of stress on patients. [3]

Although various localized radiotherapy based methods and techniques such as intraoperative radiotherapy and partial breast irradiation have been developed over the last decade, [4] available data suggests that an increase in local recurrence rates is possible. [3] Therefore, the need for new and innovative ways of eliminating residual cancer cells during or after surgery with minimal side effect can not be over emphasized.

Recently, the clinical use of heat (thermotherapy) for the treatment of cancer has received significant attention due to its minimal side effects, flexible treatment regime and potential to enhance the therapeutic efficacy of conventional cancer therapies. [5–7] Conventional techniques are based on heat sources such as radiofrequency, [8] microwave, [9] ultrasound [10] and laser. [11] However, they suffer from issues such as inadequate temperature rise and non-uniform temperature distribution within tissue. Therefore, recent efforts have focussed on externally controlled localized techniques, due to their potential to overcome the challenges associated with conventional techniques. For the last decade, the authors have explored the use of polymeric composite materials in the development of implantable biomedical device (IBD) for the post-operative single mode and multi-modal treatment of breast cancer. [12–20]

In this paper, the concepts and working principles of the devices are presented along with the results from experimental and numerical investigations are presented. The main components are a polymer matrix, a heat source and a drug-loaded thermo-sensitive gel. Here, we present two types of device concepts that differ in the type of filler used. The concept of the treatment strategy using our devices is presented in section 2. In section 4, devices based on a polymer-metal composites are presented. Nanocomposite-based devices are presented in section 5. Finally, concluding remarks and future perspectives are given in section 6.

## 2 Concept of treatment modality

**Fig 1:**
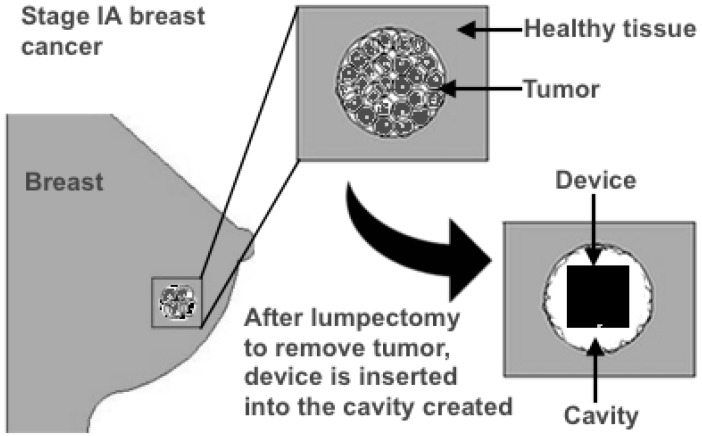
Concept of treatment modality. Schematic of how our devices could be used for post-operative treatment of cancer.

Lumpectomy is a surgical procedure performed to remove a breast lump with a surrounding margin of normal breast tissue. [21] It is also known as partial mastectomy, breast conserving surgery or wide excision. The amount of the breast removed depends on factors such as size and location of tumor. Fig 1 shows the schematic of the proposed treatment modality. It shows that the modality involves the following 3 main steps:

1. **Removal of tumor:** Lumpectomy is performed as described above.
2. **Insertion of device:** After tumor has been removed, the device is inserted into the cavity created due to the removal of the tumor after which the cut is stitched and dressed
3. **Activation of heat generation:** The mechanism of heating depends of the type of material of the heat source.

## 3 Methods

In this section, we present a brief description of the methods used to arrive at the results discussed in this paper.

### 3.1 Fabrication of devices

#### 3.1.1 Polymer-metal composites

A simple soft lithography technique was used to fabricate the PDMS matrix. First, the PDMS elastomer kit (base) and its curing agent were mixed together in a weight ratio of 10:1. This was then whisked vigorously with a spatula to produce a uniform mixture with adequate cross-linking. The resulting mixture was then placed in a desiccator for about 1 hour to ensure that the air bubbles were completely removed. Afterwards, the mixture was poured into molds containing an e-beam deposited Joule heating layer and allowed to cure at 100°C for 43 mins.

#### 3.1.2 Nanocomposites

The PDMS-based nanocomposites were prepared using a simple soft lithography technique. Nanoparticles were added to PDMS elastomer kit (base) and the mixtures were stirred thoroughly with a spatula to ensure uniform distribution of the nanoparticles and also minimize clustering. Curing agent of the PDMS base was added at a weight ratio of 10:1 and whisked vigorously with a spatula to produce a uniform mixture. The resulting mixture was then placed in a desiccator for 1 hour to completely remove air bubbles. The resulting nanocomposite mixture was poured into molds and baked at 100°C for 43 mins.

### 3.2 Material characterization

#### 3.2.1 Crystal structure

The crystal structure of the iron-oxide nano-particles was verified using a X-ray diffractometer and its size with transmission electron microscopy (TEM). The microstructure of the nanocomposite samples was studied using a scanning electron microscope.

#### 3.2.2 Magnetic properties

The magnetic properties of the nanoparticles and the nanocomposites were measured using an semi-conduction quantum interface device (SQUID) magnetometer. The magnetization curves were obtained by varying the magnetic fields between -500 mT and 500 mT at temperatures of 25°C.

#### 3.2.3 Thermometric properties

*Magnetic Nanocomposites:* Thermal characterization was carried out using an induction system (DM100) that generated an alternating magnetic field. Samples were immersed in water within a glass sample holder within the induction system. The change in the temperature of water was measured using an in-built fiber optic sensor. This was done as a function of time in the AMF. The measurements were made every 200 ms with a resolution of 0.2°C. The experimentally measured specific absorption rate (SAR_exp_) values were calculated using the expression:

$${\text{SAR}}_{\text{exp}}=\frac{\sum_{i}{\text{C}}_{\text{pi}}{\text{m}}_{\text{i}}}{{\text{m}}_{{}_{{\text{Fe}}_{3}0_{4}}}}\frac{\Delta \text{T}}{\Delta \text{t}}$$ (1)

where C_pi_ and m_i_ are the respective specific heat capacity and mass for each component (C_pi_ = 4186 J Kg^−1^ K^−1^ for water), and $m_{{}_{{\text{Fe}}_{3}0_{4}}}$ is the mass of the magnetite nanoparticles. T and t are temperature and time, respectively.

### 3.3 *In-vitro* hyperthermia studies

**Fig 2:**
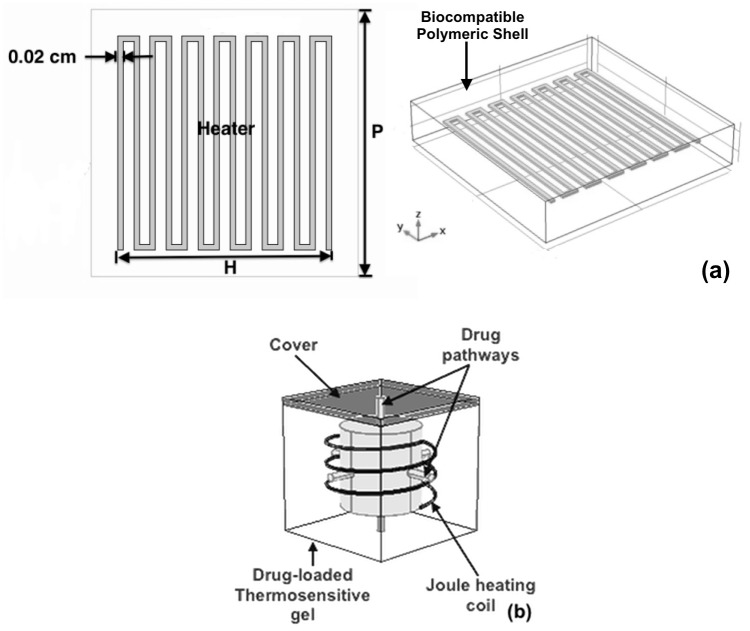
Implantable biomedical device concepts. Schematics of two different implantable biomedical devices consisting of biocompatible polymer shell with embedded (a) electrically resistive layer (heater) and (b) heater and drug-loaded thermosensitive gel.

*In-vitro* experiments using were conducted using breast cancer cells line MDA-MB-231. During the experiments, the cells are exposed to hyperthermic levels (45–48°C) using our single mode polymer-metal device (Fig 2(a)). The experiments were performed for 5, 10 and 20 cycles. The cycle populations were then analyzed using both hemocytometry and propidium staining. One-sided student’s t-test and ANOVA test were used to determine the statistical significance of the samples. A p-value lower than 0.05 was considered a significant difference and an alpha value of 0.05 was used to set the confidence interval.

## 4 Polymer-metal composites

Polymer-metal composites refer to multiphase materials in which metallic thin films are embedded within a polymer matrix. These materials have the potential to generate heat (Joule) when voltage is applied to the terminals of the thin film. Joule heating is caused when moving charges, accelerated by an applied voltage, collide with ions in the medium and give up some of their kinetic energy. The amount of heat generated depends on factors such as type and geometry of thin film material as well as the applied voltage.

### 4.1 Single mode (heat only) device

The clinical use of heat (thermotherapy) to treat cancer has been well studied. It can be applied in two main forms: hyperthermia and thermoablation. [7] In the case of hyperthermia, cancerous regions are subjected to elevated temperatures in the range between 42–46°C for durations up to 60 mins. Relatively short durations (4–15 mins) and higher temperatures (above 52°C) are used for thermoablation.

Our implant for hyperthermia treatment, shown in Fig 2(a), consists of a patterned metallic (copper) thin film (heater) embedded in a biocompatible polymer matrix (shell), poly-dimethylsiloxane (PDMS), substrate. Results of *in-vitro* hyperthermia studies obtained from this device, [14] showed that the device was able to kill or reduce the viability of breast cancer cells in the vicinity of the elevated temperature fields that surround it. Although the *in-vitro* cell experiments were promising, moving to *in-vivo* experiments required a proper understanding of the damage zones created by the device in biological media. Therfore, a combination of experiments and models was used to study the *in-vivo* temperature distribution in homogenous tissue subjected to heating by the device. The predictions from the 3D finite element method (FEM) model revealed that, a device with dimensions (polymer shell: 1 × 1 × 0.2 cm, thin layer (nichrome): 0.8 × 0.8 × 12.7 × 10^6^ cm and coil spacing: 0.04 cm), achieved hypethermic levels in about 57 s. Furthermore, it was evident from the results that size and shape of lesions could be controlled by factors such as device geometry, applied voltage and treatment time. [18] These results suggested that our device has the potential to kill post surgery residual cells within reasonable distances from the device surface.

### 4.2 Multimodal mode (chemotherapy & hyperthermia) device

Multimodal cancer therapy involves the simultaneous use of multiple cancer treatment method for a single treatment. Although the results of clinical studies have shown the potential of hyperthermia to increase efficacy when used as an adjuvant to established modalities, [22] the simultaneous use of hyperthermia and chemotherapy has been shown to have a synergistic effect that can enhance therapeutic efficiency compared to individual or sequential applications of these methods. [23]

A schematic of our device for multimodal treatment is presented in Fig 2(b). It consists of an electrically resistive layer (heater) and a drug-loaded thermosensitive gel, poly(N-isopropylacrylamide) (PNIPA), embedded within a microfabricated biocompatible polymer (PDMS) shell. We have reported different *in-vitro* simultaneous hyperthermia and drug release studies. [13, 15] As with the previous device, the conditions of hyperthermia were achieved based on Joule heating. In a proof of concept experiment, the pulsatile release of rhodamine dye from the device was compared to an unencapsulated PNIPA gel. The results showed that about 60% and 90% of the dye was released by the device and unencapsulated gel respectively after 5-min long activation (hyperthermia conditions) period every 15 min for a total of nine cycles. [13] The authors attributed the difference to the time lag associated with diffusion of drugs through the channels in the PDMS shell. Further experiments showed the time lag increases with length of the channel and flow is diffusion controlled that is affected by shape and materials in the channel. [13] Similar results were obtained using the cancer drug, biosynthesized prodigiosin. [15]

The results suggest that the proposed device concept can potentially be used for pulsatile drug delivery. The use of the biocompatible PDMS shell and incorporation of the resistive heater presents an innovative means to overcome challenges [24, 25] associated with hydrogel-based pulsatile delivery systems by exclusively releasing drugs on demand. Furthermore, the duration of drug release can be controlled by varying the length and shape of the channels in the PDMS shell as shown by our experiments. [13, 15] The current device under discussion is designed such that there will be no need for its removal after all the drugs have been released. The intention is to reuse the device in the event that the cancer cells reoccur. In such a situation, the resistive heater can be activated to achieve hyperthermic or ablative levels in the vicinity surrounding the device to cause necrosis of the cancer cells. However, it is important to note here that designs that allow the gradual degradation of the device after a period time are being explored currently.

## 5 Magnetic polymer-nanoparticle composites (nanocomposites)

Magnetic polymer-nanoparticle composities (nanocomposites) refers to multiphase composite materials in which magnetic particles with dimensions in the nano meter range (10^−9^ m) are combined with a polymer matrix. [26]

When these magnetic nanocomposites are exposed to alternating magnetic field (AMF) with given parameters (strength, *H*_0_ and frequency, *f*), the constrained magnetic NPs absorbs the magnetic energy that is dissipated as heat. The amount of heat dissipated, *A*, is equal to the area of their hysteresis loop. According to Respaud *et al.*, [27] the experimentally measured area of the hysteresis loop, *A*_exp_, can be represented as

$$A_{\text{exp}}=4\mu_{0}H_{0}\sigma_{s}\alpha$$ (2)

where *α* is the degree of optimization, which is a dimensionaless parameters that represents the ratio of the area of hysteresis loop obtained experimentally to a maximum theoretical value. *α* = 1 represents a perfectly optimized system (easy axes of all the magnetic NPs align along the AMF) and *α* = 0.39 for the case where the easy axes of the MNPs are randomly oriented. [27] In our previous studies, [17] we obtained a degree of optimization value of approximately 0.30 for our PDMS-based nanocomposites, where *μ*_0_ is the permeability of free space and *σ*_s_ is the saturation magnetization. Using *σ*_*s*_ values in our previously reported experimental data, [17, 19]*A*_exp_ is calculated for PDMS:*γ*−Fe_3_O_4_ and PDMS:Fe_3_O_4_ nanocomposites in Table 1. The results show that the heat losses increases with concentration and type of NP.

**Table 1:** Summary of magnetic and hyperthermic properties of two different nanocomposites. *A*_exp_ was calculated using Eq 2. *H*_0_ = 10 kA/m was used in the calculation of *A*_exp_.

Material Type	NP wt. % = 5		NP wt. % = 10	
	*σ*_s_ [Am^2^/kg]	*A*_exp_ [mJ/g]	*σ*_s_ [Am^2^/kg]	*A*_exp_ [mJ/g]
PDMS:Fe_3_O_4_	1.20 [17]	0.019	2.00 [17]	0.031
PDMS:*γ*−Fe_2_O_3_	2.43 [19]	0.038	3.40 [19]	0.053

### 5.1 Implantable magnetic nanocomposite thermoseeds

Localised heating using implantable magnetic thermoseeds can be considered one of the most advanced treatment modalities in terms of fundamental research and clinical research. [28] The mechanism of heat generation depends on material properties, such as the chemical composition, magnetic structure and size.

Metallic alloy thermoseeds, such as nickel-silicon, dissipate heat due to eddy currents induced by an AMF. [29] Although these kind of thermoseeds have been shown to generate high power output, issues related to biocompatibility, corrosion, fibrous encapsulation and migration across the tissues, still remain a challenge. [29]

**Fig 3:**
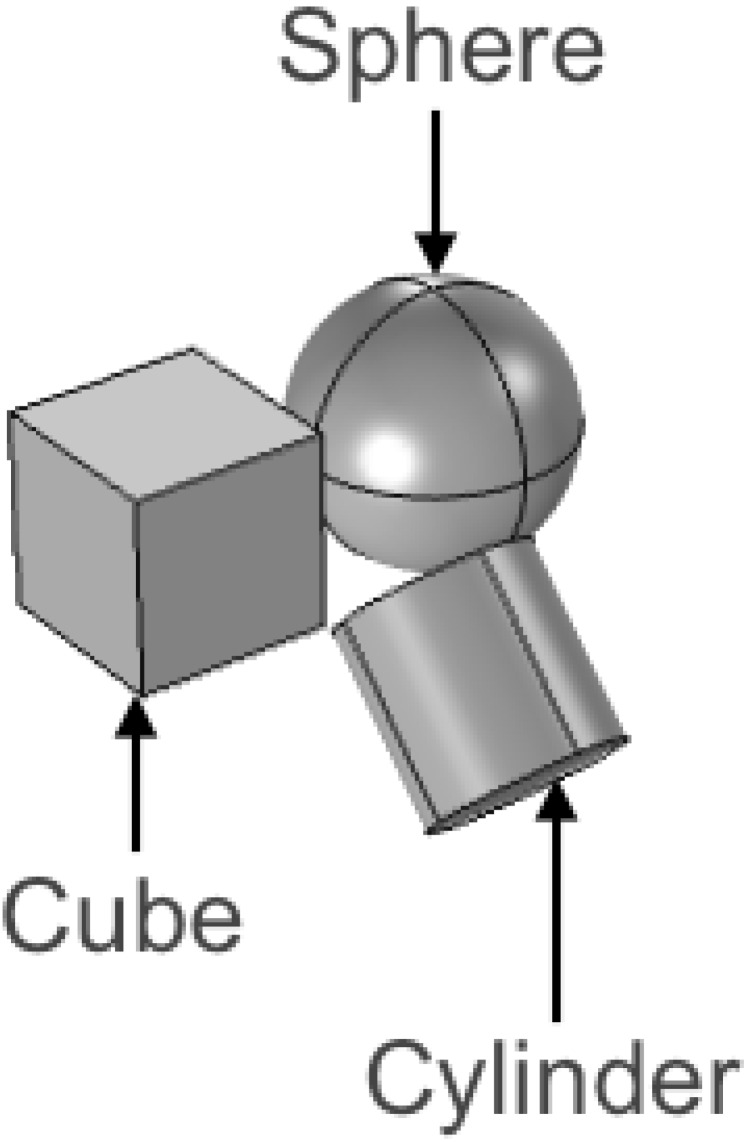
Magnetic nanocomposite thermoseeds. Schematic of various shapes of the thermoseeds, which are inserted into the cavity created after tumor is removed.

In an effort to overcome these challenges, we explored the use of nanocomposite materials in place of metallic alloys. Using a combination of experiments (water model) and models (finite element method), we simulated treatment concept described in section 2 and investigated the effects of treatment time, magnetic NP weight fraction (wt. %) and device shape (see Fig 3). [17]

Using a water model, fabricated cubic samples containing 0, 5 and 10 magnetic NP wt. % were able to increase temperature of the water by 0, 2.3 and 3.2°C, respectively for an AMF strength of 11.94 kA m^−1^ at a frequency of 298 kHz. [17] Plain PDMS (0 wt. %) was used as control. Also, a study of the dependence of specific absorption rate (SAR) on AMF strength revealed that SAR exhibit a sharp rise above the coercive field of the magnetic NPs.

Furthermore, the results from *in-vivo* predictions show that the shape of the thermal dose coverage was primarily affected by the shape of the nanocomposite for a given set of material properties, treatment time and AMF parameters. It was also revealed that the size of the thermal dose coverage was affected by both the nanocomposite geometry (size and shape) and the treatment parameters (time and temperature), for a given set of AMF parameters. Lastly, compared to other factors, the size of the nanocomposite significantly affected the volume fraction of Fe_3_O_4_ that was required to achieve a given treatment temperature.

### 5.2 Nanocomposite heating probe

Modern probe-based applicators are becoming increasingly important for thermoablation because they are localized and less invasive treatment options. The most widely used is radiofrequency ablation (RFA). [30] However, issues related to heat sink effect, charring and contact lead to decreased lesion sizes.

**Fig 4:**
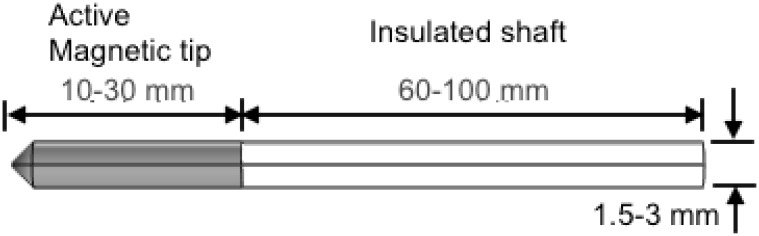
Our novel heating probe. Schematic of the magnetic heating probe. Therapeutic treatment is achieved when the active tip is brought in contact with the target area and exposed to an alternating magnetic field. Chemotherapeutic drugs can be delivered through a fabricated pathway within the probe.

Recently, we proposed a nanocomposite based probe that can potential overcome the challenges associated with conventional RFA probes and also increase efficacy. [20] Fig 4 shows a schematic of the probe, which essentially is a cannula with two main parts: a distal active tip made of a heating generating nanocomposite and a proximal electromagnetically insulated shaft. Although, the probe can be used for traditional single thermotherapy (hyperthermia or ablation), the advantage of our probe design is the potential to use it for multimodal cancer therapy involving simultaneous hyperthermia and chemotherapy.

**Fig 5:**
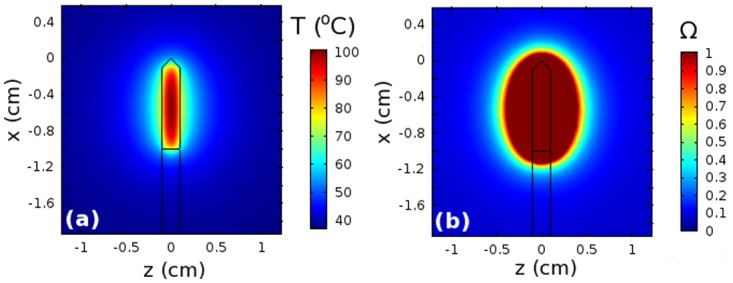
Thermal predictions. Cross-sectional (a) temperature distribution and (b) thermal damage after tissue is subjected ablation heating using a probe with a nanocomposite tip with magnetic NP volume fraction of 6.3 vol. and human-safe AMF parameters: *H*_max_ = 5 kA m^−1^ and *f* = 150 kHz.

In an effort to analyze its performance, we explored thermal damage in biological tissue subjected to localized heating by the probe. Fig 5(a) shows that the temperature distribution was non-uniform, favoring the central section of the active probe tip, where the maximum temperature occurs. The maximum temperature had no direct effect on the tissue, as it was located within the active tip. Also, we observed that the temperature was distributed symmetrically about the center of the probe tip and moved radially away from the center of the active probe tip. Fig 5(b) shows that the shape of the corresponding lesion was ellipsoidal and fairly symmetric about the probe tip. Lesion volumes of up to 2.16 cm^3^ were obtained using a probe with magnetic NP volume fraction of 6.3 vol. % and human-safe AMF parameters: *H*_max_ = 5 kA m^−1^ and *f* = 150 kHz. [20] These predictions were found to be in good agreement with previously published experimental measurements suggesting that the probe has the potential to achieve hyperthermic or ablative temperature levels using AMF parameters (*H*_max_, *f*) that are acceptable for human use.

## 6 Concluding remarks and future perspective

Polymer matrix composites offer new opportunities for the development of novel cancer treatment modalities. In this paper, the authors present concepts and working principles as well as results from experimental (*in-vitro*) and numerical investigations of the devices we have developed over the last decade. The results show the potential of the devices for localized cancer treatment.

Although, *in-vitro* experiments results from polymer-metal implants are promising, the electrochemical power source makes the device too bulky for *in-vivo* applications. This is, therefore, a need to explore light weight power sources. Within this context, wireless powering consisting of a receiver, is a viable candidate. Once attached to the implant, they can harnesses energy which can be provided by an external source. [31] This could enable the device to be remotely powered and recharged. Furthermore, using radio-frequency identification technology, temperature sensors can also be embedded in the device to provide remote and real-time monitoring of temperature and to enhance the efficacy of the treatment. [32]Alternatively, nanocomposite can replace the polymer-metal composite used in the multimodal device described in section 4.2. Clearly, further work is needed to test the drug release characteristics of such nanocomposite system under *in-vitro* and then *in-vivo* conditions.

The results obtained from *in-vivo* predictions suggest that the nanocomposite heating probes can achieve hyperthermic or ablative levels when they are exposed to human-safe AMF parameters. This provides a context within which the performance of the device could be discussed. However, the model remains a numerical model, thus errors could appear from the considerations and simplifications made to realize it. Extensive experimental work (both in-vitro and then in-vivo) is therefore needed to obtained a realistic assessment of the actual performance of the magnetic heating probe.
